# Supplementary material for: A Bayesian semi-parametric model for thermal proteome profiling
Source: Commun Biol. 2021 Jun 29;4:810. doi: 10.1038/s42003-021-02306-8 (PMC8241860; doi:10.1038/s42003-021-02306-8)
Supplement: Supplementary file 2 — Supplementary Information [file 42003_2021_2306_MOESM2_ESM.pdf]

Supplementary Information: A Bayesian semi-parametric model for thermal proteome profiling

Siqi Fang[1,4], Paul D.W. Kirk[2,3], Marcus Bantscheff[5], Kathryn S. Lilley * [1,4], and Oliver M. Crook † [1,2,4]

[1] *Cambridge Centre for Proteomics, Department of Biochemistry, University of Cambridge, Cambridge, UK*
[2] *MRC Biostatistics Unit, School of Clinical Medicine, University of Cambridge, Cambridge, UK*
[3] *Cambridge Institute of Therapeutic Immunology & Infectious Disease (CITIID), Jeffrey Cheah Biomedical Centre, Cambridge Biomedical Campus, University of Cambridge, UK.*
[4] *Milner Therapeutics Institute, Jeffrey Cheah Biomedical Centre, University of Cambridge, Cambridge, CB2 0AW, UK*
[5] *Cellzome GmbH, GlaxoSmithKline, Meyerhofstrasse 1, Heidelberg, 69117, Germany*

May 22, 2021

Supplementary Note 1 : Residual correlation matrices for TPP datasets

Panobinostat residual correlation matrix

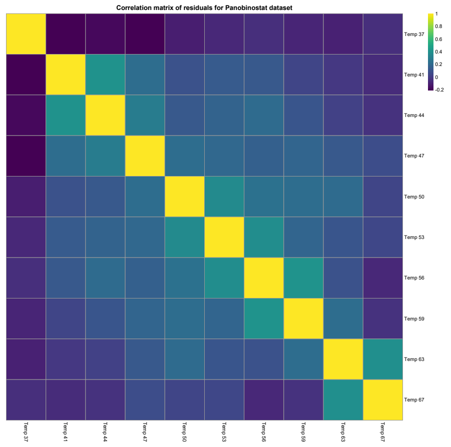

Supplementary Figure 1: Sample Spearman correlation matrix for the residuals from the sigmoid model applied to Panobinostat dataset

*ksl23@cam.ac.uk
†omc25@cam.ac.uk

Dasatinib 0.5 residual correlation matrix

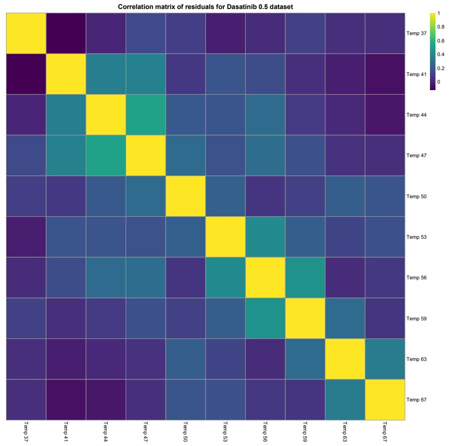

Supplementary Figure 2: Sample Spearman correlation matrix for the residuals from the sigmoid model applied to Dasatinib 0.5 dataset

Dasatinib 5 residual correlation matrix

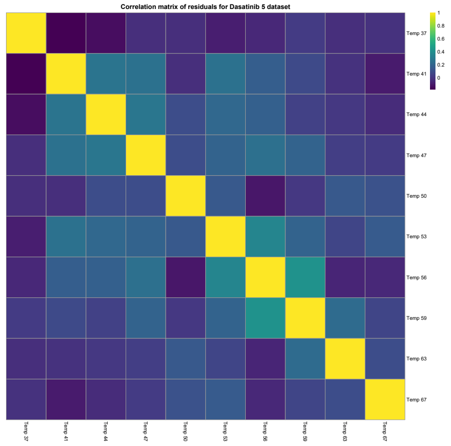

Supplementary Figure 3: Sample Spearman correlation matrix for the residuals from the sigmoid model applied to Dasatinib 5 dataset

Supplementary Note 2 : Analysis of the ATP dataset

The ATP dataset as described in the main text was also analysed to determine the sensitivity of our approaches. In this case, uniprot key-terms for "ATP-binding" were extracted and used as a pseudo ground truth. We then compute the sensitivity for each of the approaches at threshold $p = 0.01$ or posterior probability at 0.99. It is clear that the Bayesian approaches outperform the NPARC approach in this case.

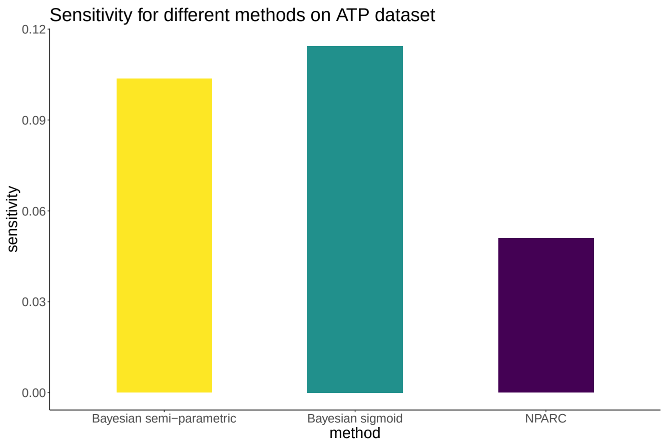

Supplementary Figure 4: Sensitivities for the different methods

Supplementary Note 3 : Simulation specification

To characterise the methods when there is a known ground truth, we perform a simulation study.

Simulation 1

Here we describe the first simulation study. We let $T = (35, 40, 45, 50, 55, 60, 65)$. The first 249 proteins are simulated form the following model, where there is no difference between the control and treatment context. Hence, for each protein i, 4 protein profiles are drawn for the following distribution

$$a_i \sim \mathcal{G}(10, 0.05) \tag{1}$$
$$b_i \sim \mathcal{U}(2, 8) \tag{2}$$
$$p_i \sim \mathcal{U}(0, 0.1) \tag{3}$$
$$x_i = S_{a,b,p}(T) = \frac{1 - p_i}{1 + \exp(b_i - \frac{a_i}{T})} + p_i \tag{4}$$
$$y_i \sim \mathcal{N}(x_i, B + \sigma I), \tag{5}$$
$$\tag{6}$$

where $\sigma = 0.01$ and $B(i, j) = \exp(-0.4(T_i - T_j)^2)$ for $i \neq j$ and 0 otherwise. The next 201 proteins are drawn from the following distribution, again with no difference between control

and treatment, with 4 profiles drawn for each protein.

$$a_i \sim \mathcal{G}(15, 0.1) \tag{7}$$
$$b_i \sim \mathcal{U}(2, 4) \tag{8}$$
$$p_i \sim \mathcal{U}(0.2, 0.3) \tag{9}$$
$$x_i = \frac{1 - p_i}{1 + \exp(b_i - \frac{a_i}{T})} + p_i \tag{10}$$
$$y_i \sim \mathcal{N}(x_i, B + \sigma I), \tag{11}$$
$$\tag{12}$$

where σ and B are as above. The above scenario is to designed to generate a null sampling population that is a mixture distribution. For cases where the treatment does have an effect on the melting profile, we simulate in the following way. For each protein i and each context $c = 1, 2$ simulate the following:

$$a_{i,c} \sim \mathcal{G}(10, 0.05) \tag{13}$$
$$b_{i,c} \sim \mathcal{U}(2, 8) \tag{14}$$
$$p_{i,c} \sim \mathcal{U}(0, 0.3) \tag{15}$$
$$x_{i,c} = \frac{1 - p_{i,c}}{1 + \exp(b_{i,c} - \frac{a_{i,c}}{T})} + p_{i,c} \tag{16}$$
$$y_{i,c} \sim \mathcal{N}(x_{i,c}, B + \sigma I), \tag{17}$$
$$\tag{18}$$

where $\sigma = 0.001$, for a total of 25 protein with 2 replicates for each context. The remaining 25 proteins are drawn from the following

$$a_i \sim \mathcal{G}(10, 0.05) \tag{19}$$
$$b_i \sim \mathcal{U}(2, 8) \tag{20}$$
$$p_i \sim \mathcal{U}(0, 0.3) \tag{21}$$
$$x_{i,1} = \frac{1 - p_i}{1 + \exp(b_i - \frac{a_i}{T})} + p_i \tag{22}$$
$$x_{i,2} = \frac{1 - p_i}{1 + \exp(b_i + \chi_1 - \frac{a_i + \chi_2}{T})} + p_i \tag{23}$$
$$\chi_1 \sim \mathcal{N}(0, 0.02) \tag{24}$$
$$\chi_2 \sim \mathcal{N}(0.1, 0.02) \tag{25}$$
$$y_{i,c} \sim \mathcal{N}(x_i, B + \sigma I), \tag{26}$$
$$\tag{27}$$

where $\sigma = 0.001$. These proteins are drawn using a different mechanism to allow flexibility in the ways the context effects the protein profiles. This simulation is designed such that the

majority of proteins are drawn from the null sampling distribution; however, there are two mechanism to generate the null and alternative models. This violate the assumptions of the NPARC model, we also note that the models are mis-specified with respect to the Bayesian models as well.

Simulation 2

Here we describe the second simulation study and we let $T = (35, 40, 45, 50, 55, 60, 65)$. The first 249 proteins are simulated form the following model, where there is no difference between the control and treatment context. Hence, for each protein i, 4 protein profiles are drawn for the following distribution

$$a_i \sim \mathcal{G}(10, 0.05) \tag{28}$$
$$b_i \sim \mathcal{U}(2, 8) \tag{29}$$
$$p_i \sim \mathcal{U}(0, 0.1) \tag{30}$$
$$x_i = S_{a,b,p}(T) = \frac{1 - p_i}{1 + \exp(b_i - \frac{a_i}{T})} + p_i \tag{31}$$
$$y_i \sim \mathcal{N}(x_i, B + \sigma I), \tag{32}$$
$$\tag{33}$$

where $\sigma = 0.01$ and $B(i,j) = \exp(-0.4(T_i - T_j)^2)$ for $i \neq j$ and 0 otherwise. For the next 101 proteins are drawn from the following, again with no difference between control and treatment, with 4 profiles drawn for each protein.

$$a_i \sim \mathcal{G}(15, 0.1) \tag{34}$$
$$b_i \sim \mathcal{U}(2, 4) \tag{35}$$
$$p_i \sim \mathcal{U}(0.2, 0.3) \tag{36}$$
$$x_i = \frac{1 - p_i}{1 + \exp(b_i - \frac{a_i}{T})} + p_i \tag{37}$$
$$y_i \sim \mathcal{N}(x_i, B + \sigma I), \tag{38}$$
$$\tag{39}$$

where σ and B are as above. The above scenario is to designed to generate a null sampling population that is a mixture distribution. For cases where the treatment does have an effect on the melting profile, we simulate in the following way. For each protein i and each context

$c = 1, 2$ simulate the following:

$$a_{i,c} \sim \mathcal{G}(10, 0.05) \tag{40}$$
$$b_{i,c} \sim \mathcal{U}(2, 8) \tag{41}$$
$$p_{i,c} \sim \mathcal{U}(0, 0.3) \tag{42}$$
$$x_{i,c} = \frac{1 - p_{i,c}}{1 + \exp(b_{i,c} - \frac{a_{i,c}}{T})} + p_{i,c} \tag{43}$$
$$y_{i,c} \sim \mathcal{N}(x_{i,c}, B + \sigma I), \tag{44}$$
$$\tag{45}$$

where $\sigma = 0.001$, for a total of 100 protein with 2 replicates for each context. The remaining 50 proteins are drawn from the following

$$a_i \sim \mathcal{G}(10, 0.05) \tag{46}$$
$$b_i \sim \mathcal{U}(2, 8) \tag{47}$$
$$p_i \sim \mathcal{U}(0, 0.3) \tag{48}$$
$$x_{i,1} = \frac{1 - p_i}{1 + \exp(b_i - \frac{a_i}{T})} + p_i \tag{49}$$
$$x_{i,2} = \frac{1 - p_i}{1 + \exp(b_i + \chi_1 - \frac{a_i + \chi_2}{T})} + p_i \tag{50}$$
$$\chi_1 \sim \mathcal{N}(0.1, 0.02) \tag{51}$$
$$\chi_2 \sim \mathcal{N}(0.1, 0.02) \tag{52}$$
$$y_{i,c} \sim \mathcal{N}(x_i, B + \sigma I), \tag{53}$$
$$\tag{54}$$

where $\sigma = 0.001$. These proteins are drawn using a different mechanism to allow flexibility in the ways the context effects the protein profiles. This simulation is designed such that only 70% of proteins are drawn from the null sampling distribution and there are two mechanism to generate the null and alternative models. These violate the assumptions of the NPARC model, we also note that the models are mis-specified with respect to the Bayesian models as well.

Simulation 3

The same for simulation study 1, but the prior on the length scale is change to $\mathcal{LN}(1, 0.5)$ to test the sensitivity of the analysis.

Supplementary Note 4: Simulation study results

This section reports the simulation study results. All approaches are capable of controlling the FDR and, as we observed for real data, the Bayesian models show improved sensitivity.

Since the simulation temperatures are at different increments to the real data, the default priors are changed to $a \sim \mathcal{G}(7, 0.07)$ and $l \sim \mathcal{LN}(0.5, 0.5)$. The other priors are held the same.

Simulation 1

The simulation study 1 is performed 10 times to report a distribution of results. We report the distribution of sensitivity at a fixed specificity of 0.99 for all methods (figure 5). We also plot an example ROC curve from these simulations in figure 6. It is clear that the Bayesian models shows improvement over NPARC and that the semi-parametric model is an improvement over the sigmoid models.

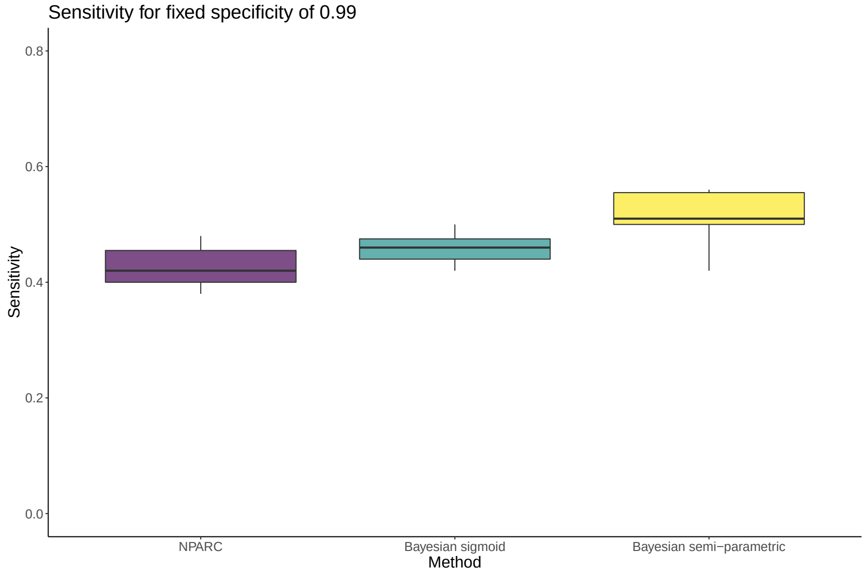

Supplementary Figure 5: Sensitivities for a fixed specificity of 0.99. The boxplots are distributions from repeated simulations.

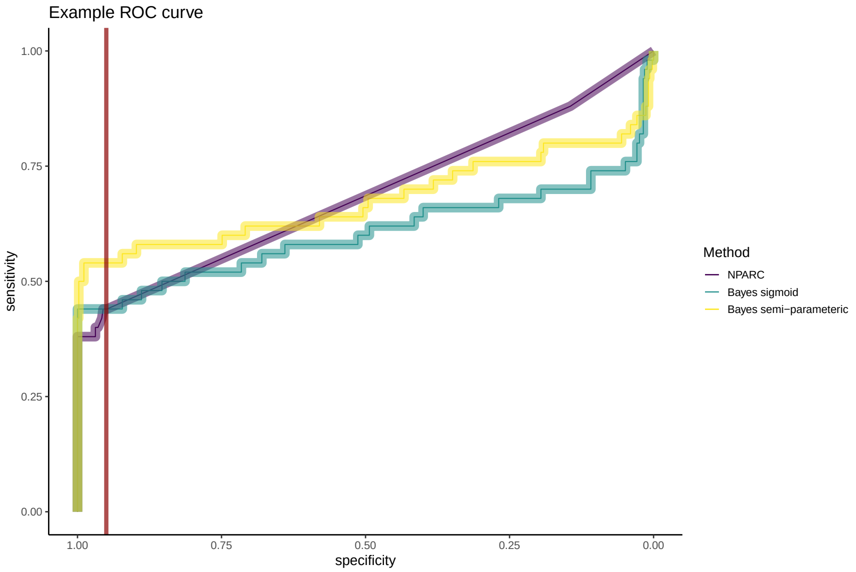

Supplementary Figure 6: An example ROC curve from the simulations. The dark red line indicates a specificity of 0.95

Simulation 2

The simulation study 2 is performed 10 times to report a distribution of results. We report the distribution of sensitivity at a fixed specificity of 0.99 for all methods (figure 7). We also plot an example ROC curve from these simulations in figure 8. It is clear that the Bayesian models shows improvement over NPARC and that the semi-parametric model is an improvement over the sigmoid models.

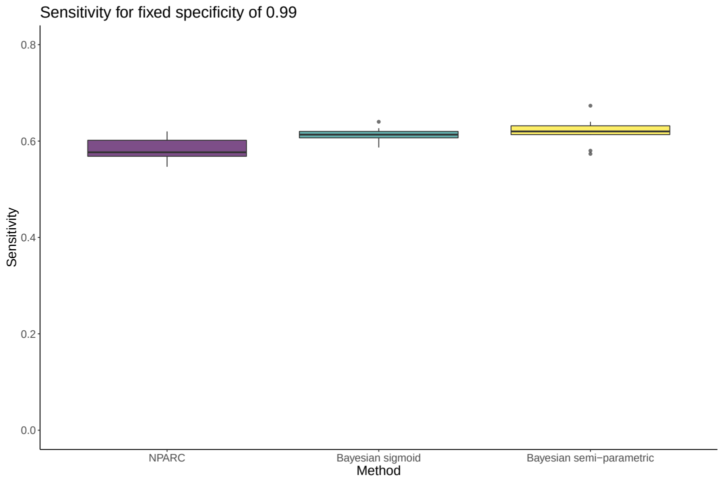

Supplementary Figure 7: Sensitivities for a fixed specificity of 0.99. The boxplots are distributions from repeated simulations.

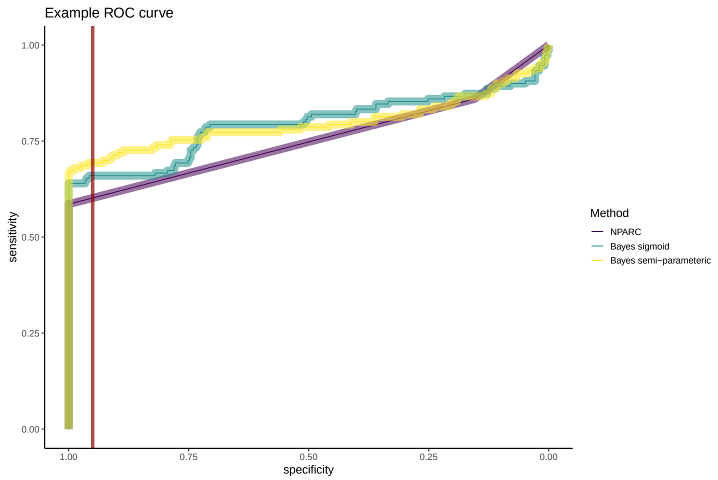

Supplementary Figure 8: An example ROC curve from the simulations. The dark red line indicates a specificity of 0.95

Simulation 3

The simulation study 3 is performed 10 times to report a distribution of results. We report the distribution of sensitivity at a fixed specificity of 0.99 for all methods (figure 9). We also plot an example ROC curve from these simulations in figure 10. It is clear that the Bayesian models shows improvement over NPARC and that the semi-parametric model is an improvement over the sigmoid models.

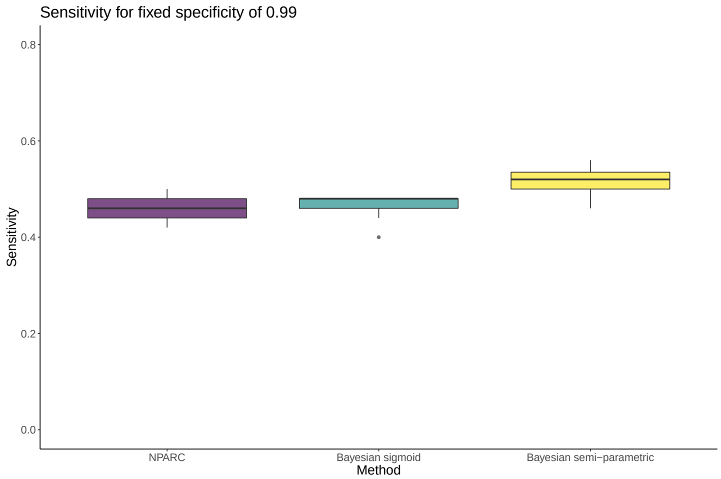

Supplementary Figure 9: Sensitivities for a fixed specificity of 0.99. The boxplots are distributions from repeated simulations.

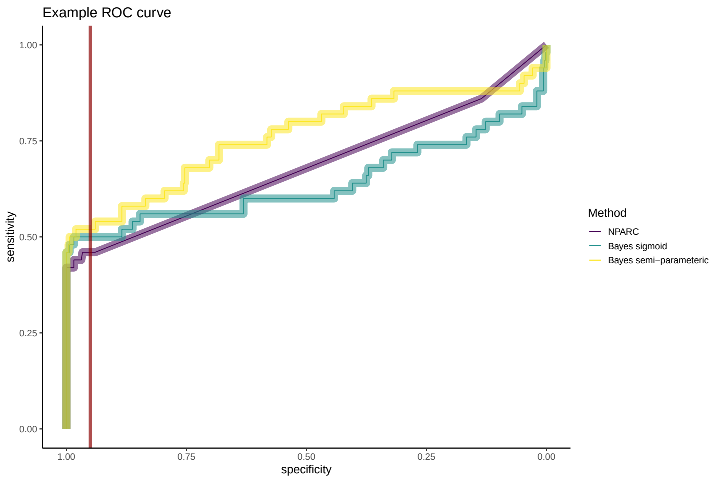

Supplementary Figure 10: An example ROC curve from the simulations. The dark red line indicates a specificity of 0.95

Supplementary Note 5: Model selection

In this section, we further evaluate the Bayesian sigmoid model and Bayesian semi-parametric approach. We examine the marginal likelihood, averaged over proteins and report the distribution over simulations. Figure 11 shows that the marginal likelihood for the semi-parametric is considerably higher than the Bayesian sigmoid model. We also evaluate out-of-sample pre-

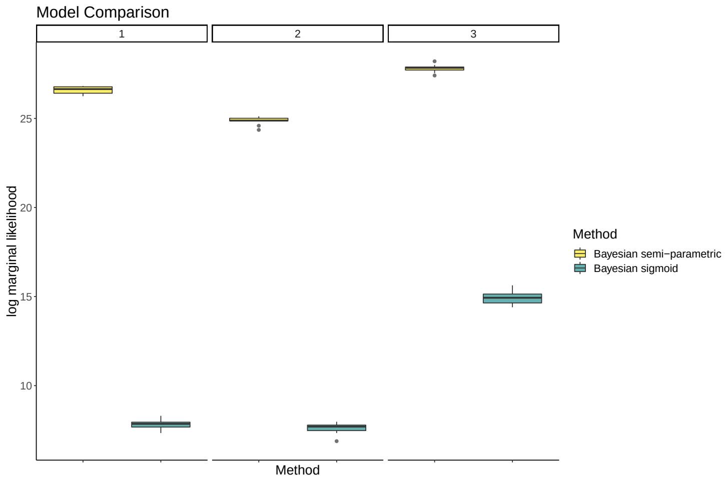

Supplementary Figure 11: Marginal likelihoods for the different Bayesian models where distributions are over different simulations.

dictive performance for both Bayesian models. We examine the leave-one-out cross-validation estimate with log scoring. Figure 12 shows that the Bayesian sigmoid model has higher expected log predictive density (averaged over proteins). This is perhaps antagonistic with the log marginal likelihood; however, it is well documented that non-parametric models can reduce out-of-sample predictive performance (?). That being said the improvement is less than 2 points.

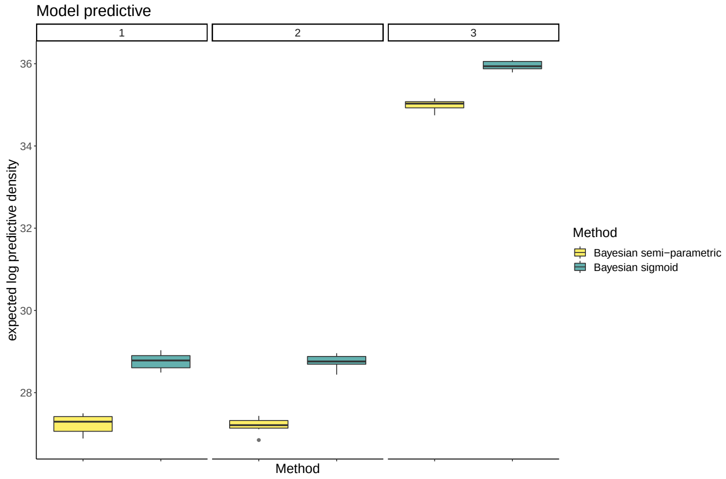

Supplementary Figure 12: LOO-CV estimates of the expected log predictive density, averaged over proteins. The distributions are over different simulations

Supplementary Note 6: Robustness to model miss-specification

In this section, we test robustness of the method to model miss-specification. To be more precise, we consider a simulation scenario where the model is not from the class of models being fit. Recall simulation scenario 1 and instead of simulating data from the 3-parameter sigmoid model, we simulate for the following 4-parameter sigmoid model:

$$S_{a,b,p,q} = \frac{1-p}{(1+\exp(b-\frac{a}{T}))^q} + p \tag{55}$$

Note that in this 4-parameter sigmoid model, a degree of asymmetry is allowed. We set $q = 10$ for this simulation and apply NPARC and the Bayesian model. The simulations are performed 10 times to generate a distribution of results. Fortunately, both the Bayesian models are robust to this model miss-specification and are able to successfully control the false positive rate and have high sensitivity (see figures 13 and 14). NPARC is much more sensitive to model miss-specification.

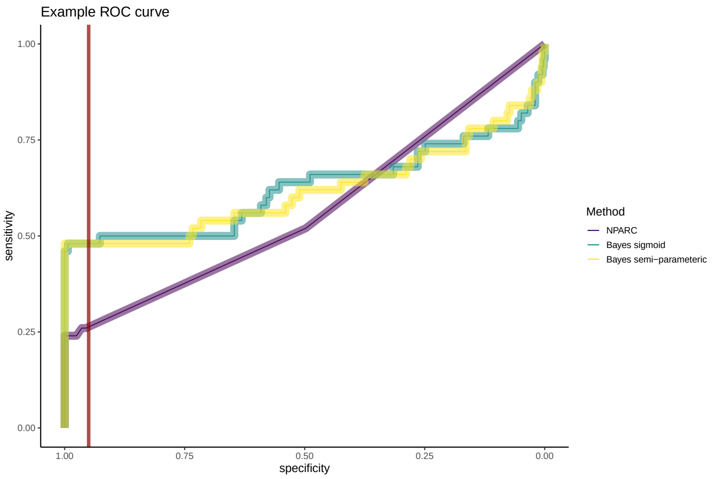

Supplementary Figure 13: An example ROC curve from the miss specified model. The dark red line indicates a specificity of 0.95

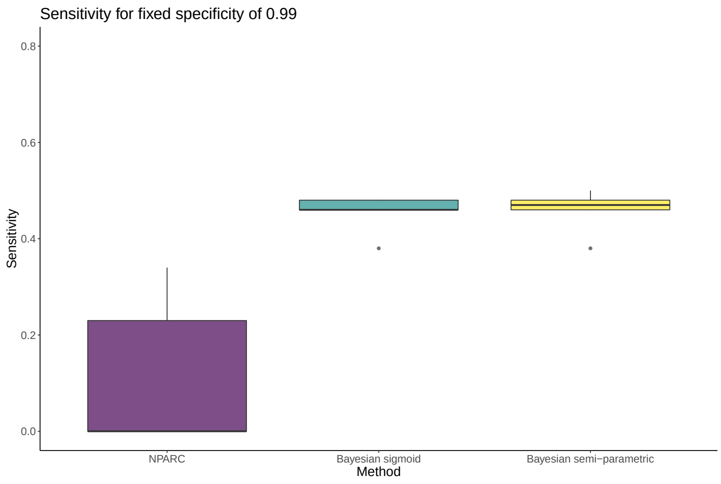

Supplementary Figure 14: Sensitivities for a fixed specificity of 0.99 for miss specified model. The boxplots are distributions from repeated simulations.

Supplementary Note 7: Posterior predictive checks

To complement the posterior predictive checks in the main text for AP4S1. We simulate 100 datasets y_{rep} from the posterior predictive distribution of each of the two Bayesian models. For each dataset, we compute the kernel density estimate of the simulated datasets, as well as the observed data. We plot the results for the sigmoid model in figure 15 and the semi-parametric model in figure 16. It is evident that the posterior predictive check for the semi-parametric model is more appropriate for the observed data.

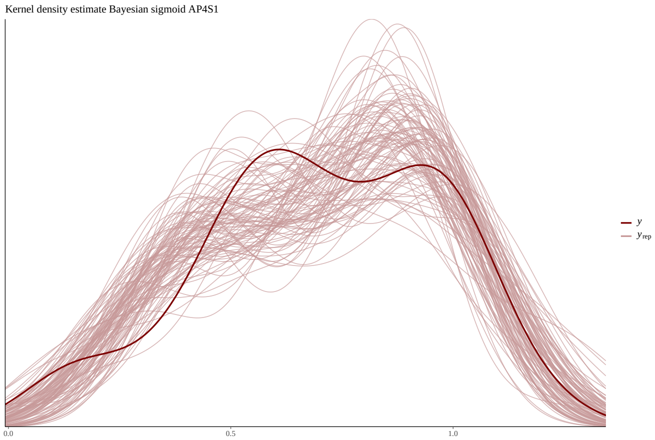

Supplementary Figure 15: Kernel density estimate posterior predictive check for sigmoid model

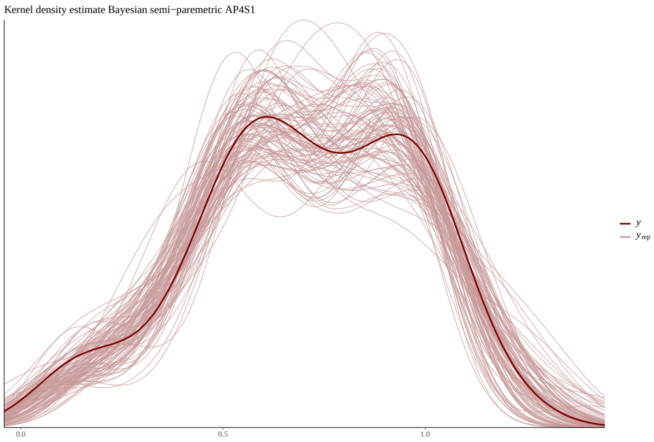

Supplementary Figure 16: Kernel density estimate posterior predictive check for semi-parametric model
