# Supplementary material for: A Bayesian semi-parametric model for thermal proteome profiling
Source: Commun Biol. 2021 Jun 29;4:810. doi: 10.1038/s42003-021-02306-8 (PMC8241860; doi:10.1038/s42003-021-02306-8)
Supplement: Supplementary file 3 — Description of Additional Supplementary Files [file 42003_2021_2306_MOESM3_ESM.pdf]

Description of Additional Supplementary Files

File name: Supplementary Data 1

Description: Posterior Probabilities of both models for each of the 5 datasets.

File name: Supplementary Data 2

Description: Proteins with 5% more variance explained using the Bayesian semi-parametric model than the sigmoid model.

File name: Supplementary Data 3

Description: Data for Uniprot enrichment results and intrinsically disordered regions.

File name: Supplementary Data 4

Description: Data underlying the figures in the text.

File name: Supplementary Code

Description: Stan files for null and alternative models for sigmoid and semi-parametric models.
